# Supplementary material for: Development and validation of the Cannabis Exposure in Pregnancy Tool (CEPT): a mixed methods study
Source: BMC Pregnancy Childbirth. 2024 Apr 16;24:280. doi: 10.1186/s12884-024-06485-0 (PMC11022340; doi:10.1186/s12884-024-06485-0)
Supplement: Supplementary file 1 — Supplementary Material 1. [file 12884_2024_6485_MOESM1_ESM.pdf]

Appendix A: Cannabis Exposure in Pregnancy Tool (CEPT) - print rendering of electronic tool
* notes in red do not appear in user version

CEPT - Cannabis Exposure in Pregnancy Tool

We understand that some people choose to use cannabis in pregnancy, and we would like to learn more about them. We want to help ensure that everyone receives the best prenatal care possible.
Please answer about your current pregnancy only. If you are not pregnant, answer about your most recent pregnancy.

1. How many weeks pregnant are you today? {dropdown of weeks} ○ I am not currently pregnant

2. During your current/most recent pregnancy how often would you say you were exposed to second-hand *cannabis* smoke or *cannabis* vapour while in the same room as the person using it? ***if never, skip to 4**

Never	A few times, but less than once per month	About monthly	About weekly	Daily or almost daily	Multiple times per day
○	○	○	○	○	○

3. If you were exposed to second hand *cannabis* smoke or *cannabis* vapour while talking or hanging out with the person using, at what time(s) in your current/most recent pregnancy were you exposed? (select all that apply)

1st Trimester	2nd Trimester	3rd Trimester	Unsure
○	○	○	○

4. At what time(s) in your current pregnancy, so far, did you use cannabis or cannabis products?(Select all that apply).
***if 'didn't use', skip to 7 and END**

I didn't use cannabis	I quit when I found out I was pregnant coded as 1st Tri.	1st Trimester	2nd Trimester	3rd Trimester
○	○	○	○	○

5. During you your (indicate trimester from item 4) how often would you say you used cannabis or cannabis products for either medical or non-medical purposes?
*Item 5 repeats for each period indicated in item 4

Once or twice	A few times, but less than once per month	About monthly	About weekly	Daily or almost daily	Multiple times per day
○	○	○	○	○	○

6. During your current/most recent pregnancy, if you used any cannabis or cannabis products, it was mainly:

- ○ To help with pain
- ○ For enjoyment
- ○ To help with morning sickness/nausea
- ○ To help with appetite
- ○ To relax
- ○ For seizures
- ○ To help with sleep
- ○ To help with depression
- ○ Other (please specify)
- ○ To help with anxiety

7. Does your partner consume cannabis or cannabis products?

○ I don't currently have a partner ○ Yes, sometimes ○ Yes, regularly ○ No

8. During your current/most recent pregnancy if you used cannabis or cannabis products, in what form did you use them?

- ○ Smoked dried buds/flowers/leaves (Pipe and/or joints etc)
- ○ Vaped
- ○ Ate or drank products with THC (edibles, mist, tinctures, capsules or pills)
- ○ Ate or drank products with CBD only (edibles, mist, tinctures, capsules or pills)
- ○ Applied skin products with THC (cream, patch, lip-balm etc.)
- ○ Applied skin products with CBD only (cream, patch, lip-balm etc.)
- ○ Used shatter, budder, hash, oil, rosin or other concentrated product
- ○ Other (please specify)

The next questions ask about how much cannabis or cannabis product you use at a time. Please select the amounts that correspond to each type of cannabis product you said you used in the last question.

Answer separately for each type of cannabis product, indicating your best guess of how much you would use at one time.

*Items 9a-9i appear for only those categories selected in item 8

9a. If you smoked dried cannabis, how much would you say you smoked each time? (your best guess is okay)

- ○ 1 to less than 5 inhalations/puffs
- ○ 5 inhalations to less than 1/8 gram (pea-sized)
- ○ 1/8 gram to less than 1/4 gram (blueberry-sized)
- ○ 1/4 gram to less than 1/2 gram (grape-sized)
- ○ 1/2 gram to less than 1 gram (1- 1.5 grapes)
- ○ 1 gram or more

9b. If you vaped cannabis bud or juice, *not concentrate/distillate/budder/hash/oil* how much would you say you vaped each time? (your best guess is okay)

- ○ 1 or 2 inhalations (puffs)
- ○ 3 inhalations (puffs) to less than 5 inhalations/puffs
- ○ 5 inhalations/puffs to less than 1/8 cartridge/pen
- ○ 1/8 to less than 1/4 cartridge/pen
- ○ 1/4 cartridge/pen to less than 1/2 cartridge/pen
- ○ 1/2 cartridge/pen or more

**9c. If you ate or drank cannabis product containing <u>THC</u> (i.e. edibles, tea/drinks, mist, pill, capsule), how much would you say you consumed each time? (your best guess is okay)**	Less than 10mg THC ○	10 to 30 mg THC ○	31 to 60 mg THC ○	61 to 120 mg THC ○	Over 120mg THC ○
**9d. If you ate or drank cannabis product containing <u>CBD only</u> (i.e. edibles, tea/drinks, mist, pill, capsule), how much did you consume each time? (your best guess is okay)**	Under 10mg CBD ○	10 to 30 mg CBD ○	31 to 60 mg CBD ○	61 to 120 mg CBD ○	Over 120mg CBD ○
**9e. If you used topical cannabis products containing <u>THC</u> (i.e. skin creams or oils, patches, etc.), how much did you use each time? (your best guess is okay)**	Dime-sized amount ○	Nickel-sized amount ○	Quarter-sized amount ○	Loonie-sized amount ○	More than a Loonie-sized amount ○
**9f. What percentage THC is the skin product you used?**	less than 10% ○	10% to 15% ○	16% to 25% ○	26% to 35% ○	More than 35% ○
**9g. If you used topical <u>CBD only</u> products (i.e. skin creams, patches, balms, etc.), how much would you say you used each time? (your best guess if okay)**	Dime-sized amount ○	Nickel-sized amount ○	Quarter-sized amount ○	Loonie-sized amount ○	More than a Loonie-sized amount ○
**9h. What percentage CBD is the skin product you used?**	less than 10% ○	10% to 15% ○	16% to 25% ○	26% to 35% ○	More than 35% ○
**9i. If you used shatter, budder, hash, oil, rosin or other concentrated products, how much would you say you used each time? (your best guess is okay)**	Less than 1/8 gram (1-3 rice grains) ○	1/8 - 1/4 to gram (mini M&M) ○	More than 1/4g. (mini M&M) ○		
