# Supplementary material for: Development and validation of the Cannabis Exposure in Pregnancy Tool (CEPT): a mixed methods study
Source: BMC Pregnancy Childbirth. 2024 Apr 16;24:280. doi: 10.1186/s12884-024-06485-0 (PMC11022340; doi:10.1186/s12884-024-06485-0)
Supplement: Supplementary file 2 — Supplementary Material 2. [file 12884_2024_6485_MOESM2_ESM.docx]

Supplemental Fig 1: Sample characteristics versus maternal population of Canada
